# Infodemia: noticias falsas y tendencias de mortalidad por COVID-19 en seis países de América Latina

**DOI:** 10.26633/RPSP.2021.44

**Published:** 2021-05-13

**Authors:** Giselly Mayerly Nieves-Cuervo, Edgar F. Manrique-Hernández, Angelo Fernando Robledo-Colonia, Elvia Karina Grillo Ardila

**Affiliations:** 1 Escuela de Salud Pública, Facultad de Salud Universidad del Valle Cali Colombia Escuela de Salud Pública, Facultad de Salud, Universidad del Valle, Cali, Colombia.; 2 Departamento de Salud Pública, Escuela de Medicina Universidad Industrial de Santander Bucaramanga Colombia Departamento de Salud Pública, Escuela de Medicina, Universidad Industrial de Santander, Bucaramanga, Colombia.; 3 Escuela de Salud, Facultad de Salud Universidad del Valle Cali Colombia Escuela de Salud, Facultad de Salud, Universidad del Valle, Cali, Colombia.

**Keywords:** Comunicación en salud, acceso a la información, infecciones por coronavirus, redes sociales en línea, América Latina, Health communication, access to information, coronavirus infections, online social networking, Latin America, Comunicação em saúde, acesso à informação, infecções por coronavirus, redes sociais online, América Latina

## Abstract

**Objetivo.:**

Describir el comportamiento de la diseminación de noticias falsas en el contexto de la mortalidad por COVID-19 y el manejo de la infodemia en seis países latinoamericanos.

**Métodos.:**

Estudio ecológico descriptivo que explora el porcentaje de la población con incapacidad para reconocer las noticias falsas, el porcentaje de confianza en el contenido de las redes sociales y el porcentaje de su uso como única fuente de noticias en Argentina, Brasil, Chile, Colombia, México y Perú hasta el 29 de noviembre del 2020. Se calculó el índice de penetración de Internet en cada país, la tasa de penetración de Facebook y la tasa de mortalidad por la COVID-19. La información sobre las medidas implementadas se obtuvo mediante búsquedas bibliográficas y en portales gubernamentales y de noticias de los países seleccionados, según las cinco áreas de acción propuestas por la Organización Mundial de la Salud: identificación de la evidencia, trasmisión de la ciencia y el conocimiento, acciones amplificadas, cuantificación del impacto, y coordinación y gobernanza.

**Resultados.:**

Chile y Argentina fueron los países con los mayores índices de penetración de Internet (92,4% y 92,0%, respectivamente) y también están entre los que mayor uso hacen de las redes sociales como único medio para la obtención de noticias (32,0% y 28,0%, respectivamente); Brasil y Colombia mostraron un comportamiento intermedio en ambos indicadores. México tiene el uso más alto de redes sociales, mientras Perú y Colombia presentaron los mayores valores del índice de incapacidad para reconocer noticias falsas.

**Conclusiones.:**

Se observó que en los países con menor uso de las redes sociales como único medio para la obtención de la información y menor confianza en el contenido de redes sociales, las tasas de mortalidad fueron también menores.

## Abstract (en)

**Objective.:**

Describe patterns in the dissemination of fake news in the context of COVID-19 mortality and infodemic management in six Latin American countries.

**Methods.:**

A descriptive ecological study explored the percentage of the population that is unable to recognize fake news, the percentage who trust social network content, and the percentage who use it as their sole news source in Argentina, Brazil, Chile, Colombia, Mexico, and Peru, up to 29 November 2020. Internet penetration rate, Facebook penetration rate, and COVID-19 mortality were calculated for each country. Information was obtained from literature searches and government and news portals in the selected countries, according to the World Health Organization’s five proposed action areas: identifying evidence, translating knowledge and science, amplifying action, quantifying impact, and coordination and governance.

**Results.:**

Chile and Argentina were the countries with the greatest internet penetration rates (92.4% and 92.0%, respectively) and were also among the heaviest users of social media as their only means of obtaining news (32.0% and 28.0%, respectively). Brazil and Colombia showed intermediate behavior for both indicators. Mexico had the highest use of social networks, while Peru and Colombia had the highest indices of inability to recognize fake news.

**Conclusions.:**

It was observed that in countries with less use of social networks as the sole means for obtaining information and less trust in social network content, mortality was also lower.

## RESUMO

**Objetivo.:**

Descrever o comportamento da disseminação de notícias falsas no contexto da mortalidade por COVID-19 e controle da infodemia em seis países latino-americanos.

**Métodos.:**

Estudo ecológico descritivo que explora o percentual da população com dificuldade de identificar notícias falsas, o percentual de confiança no conteúdo das redes sociais e o percentual de uso das redes como única fonte de notícias na Argentina, Brasil, Chile, Colômbia, México e Peru até 29 de novembro de 2020. Foram calculados o índice de penetração da internet, o índice de penetração do Facebook e a taxa de mortalidade por COVID-19 em cada país. Os dados foram obtidos por meio de pesquisas bibliográficas e em sites do governo e de notícias dos países selecionados segundo as cinco áreas de ação propostas pela Organização Mundial da Saúde (OMS): identificar a evidência, transmitir ciência e conhecimento, amplificar ações, mensurar o impacto e coordenação e governança.

**Resultados.:**

O Chile e a Argentina foram os países com os maiores índices de penetração da internet (92,4% e 92,0%, respectivamente) e estão entre os países com maior uso das redes sociais como única fonte de notícias (32,0% e 28,0%, respectivamente). O Brasil e a Colômbia tiveram um comportamento intermediário em ambos os indicadores. O México tem o maior uso das redes sociais e o Peru e a Colômbia apresentaram os maiores índices de dificuldade de identificar notícias falsas.

**Conclusões.:**

Observou-se que, nos países com menor uso das redes sociais como única fonte para obter informação e menor confiança no conteúdo das redes sociais, a taxa de mortalidade também foi menor.

Las tecnologías digitales de la información han adquirido un papel preponderante durante la actual pandemia de COVID-19, causada por el virus SARS-CoV-2. En América Latina, el avance y la velocidad de implementación de estos recursos ha sido menor en comparación con los países industrializados, y su impacto no es homogéneo en la subregión (1). El monitoreo y la vigilancia de la salud púbica, el seguimiento de los casos y sus contactos, y la evaluación de las intervenciones y los procedimientos empleados en la población han permitido avanzar en el conocimiento y dar respuestas más rápidas para el control de la enfermedad. Sin embargo, el acceso a grandes volúmenes de datos en línea —muchas veces sin la suficiente validación— ha generado, de manera paralela, dudas sobre la calidad y la fiabilidad de la información disponible en temas de salud (2).

Las tecnologías digitales son herramientas de gran alcance y utilidad en la salud pública; no obstante, para lograr el resultado esperado es indispensable garantizar el manejo responsable de la información y velar por su buena calidad, a fin de evitar la difusión —masiva o no— de noticias y rumores falsos (incluidos los engañosos e inexactos) y el predominio de medios poco confiables (3). La actual emergencia ha tenido una gran repercusión sanitaria, social, económica y política; en este contexto, la diseminación masiva (para lo cual se ha acuñado el término “viralización”) de la información falsa ha actuado de manera directa y colectiva sobre los lectores (4).

El enorme volumen de información generado por la pandemia se ha propagado tanto por medios de comunicación tradicionales como digitales, lo que hace difícil encontrar fuentes confiables y seguras cuando se las necesita (5). La información disponible sobre la pandemia ha aumentado de manera exponencial entre el 50% y el 70% (6), cifras que incluyen en gran medida la desinformación —la propagación de rumores y la manipulación con intenciones dudosas—, un fenómeno amplificado por medio de las redes sociales (2).

En respuesta a esta situación, surgió la infodemiología, una nueva disciplina reconocida por la Organización Mundial de la Salud (OMS) y otras organizaciones de salud pública, que se enfoca en cuatro vertientes: a) el fomento de la alfabetización científica y en temas de salud, b) los procesos de perfeccionamiento del conocimiento y la mejora de su calidad, c) la verificación de datos y la revisión por pares, y d) la traducción precisa y oportuna de conocimientos, sin distorsiones o influencias comerciales o políticas. Estas líneas de trabajo se han convertido en los cuatro pilares de la infovigilancia, entendida como la gestión de infodemias en épocas de crisis, como la debida a la pandemia por COVID-19 (7).

Según la OMS, ya en marzo del 2020 se utilizaban los términos “coronavirus” y “pandemia”, o similares, en más de 550 millones de tuits y para el mes de abril se habían publicado en Internet cerca de 360 millones de videos con los términos “COVID 19” y “COVID-19” en sus etiquetas (2). La circulación de mensajes, audios y videos con noticias no confiables relacionadas con la pandemia —incluidas afirmaciones sobre la inexistencia del virus— ocasionaron que un segmento de la población decidiera no hacer caso a las medidas preventivas y se diseminara a gran escala en la población la angustia y el temor —o la indiferencia— ante esta enfermedad. Así mismo, la proliferación de noticias falsas sobre aparentes curas y tratamientos contra la COVID-19 puso en riesgo la salud de muchas personas (8).

Se ha documentado que el 33% de las personas de América Latina, en promedio, obtiene información diaria a través de redes sociales, mientras solo el 17% utiliza medios más tradicionales (9). Debido a las implicaciones de las comunicaciones en la adopción de comportamientos por parte de sus consumidores y de su impacto sobre las acciones en salud pública, plataformas como Google, Twitter y Facebook implementaron estrategias para evitar la propagación de noticias falsas, como el direccionamiento durante la búsqueda de información a contenidos de páginas gubernamentales y de autoridades sanitarias (10).

Por todo lo anterior, la infodemiología se ha constituido como una herramienta útil para el estudio de la sobreabundancia de información, que puede llevar a la diseminación de noticias falsas, engañosas e inexactas sobre la actual pandemia. Este estudio tiene como objetivo describir el comportamiento de la diseminación de noticias falsas en el contexto de la mortalidad por COVID-19 y el manejo de la infodemia en seis países latinoamericanos seleccionados.

## MATERIALES Y MÉTODOS

Se realizó un estudio ecológico descriptivo, en el que la unidad de análisis fueron seis países latinoamericanos: Argentina, Brasil, Chile, Colombia, México y Perú; el marco temporal fue desde el inicio de la pandemia por COVID-19 en cada territorio (cuadro 1) hasta el 29 de noviembre del 2020. Se decidió utilizar el enfoque ecológico —a pesar de sus limitaciones para hacer inferencias individuales y causales, y sus posibles sesgos (33)— como un acercamiento para explorar la infodemia en cada país y generar hipótesis sobre este tema emergente. Los países se seleccionaron por la disponibilidad de información relacionada con el objetivo de esta investigación, la gran afectación por la COVID-19 y el alto grado de universalización del acceso a las tecnologías digitales en ellos.

### Análisis descriptivo

Teniendo en cuenta las características de la diseminación de las noticias falsas en la actual pandemia, se exploraron tres aristas: el porcentaje de la población con incapacidad para reconocer las noticias falsas, el porcentaje de confianza en el contenido de las redes sociales y el porcentaje de su uso como única fuente de noticias a diario. La información de las variables descritas anteriormente se obtuvo de la encuesta realizada por Kaspersky y Corpa en febrero del 2020 (34).

Para ello, se calculó el índice de penetración de Internet en cada país, definido como la proporción de hogares con al menos un miembro de entre 16 y 74 años de edad con acceso a Internet (expresado como porcentaje). Para el análisis de las redes sociales se utilizó la tasa de penetración de Facebook, definida como el número de usuarios de esa red entre el total de la población de cada país (expresado como porcentaje); se escogió Facebook debido al elevado número de usuarios y la disponibilidad de información en los países evaluados.

La cantidad de personas con acceso a Internet y de usuarios de Facebook, así como los datos de noticias falsas sobre COVID-19 publicadas en Youtube, Twitter y Whatsapp, se calcularon a partir de la información ofrecida por Internet World Stats (35) para cada país en el año 2020. Se calculó la tasa de mortalidad por COVID-19 por 100 000 habitantes hasta el 29 de noviembre en cada país, a partir de los datos de la OMS de mortalidad por la COVID-19 y el total de la población por países, según su actualización del día 29 de noviembre del 2020 (36).

Los datos se tabularon en Excel® y se utilizó el paquete estadístico STATA™, versión 15, para el análisis exploratorio de los datos y el cálculo de las tasas.

### Análisis de las medidas implementadas

Se realizó una búsqueda de artículos publicados referenciados en Pubmed y SciELO, y la literatura gris disponible en los portales gubernamentales y de noticias de los países seleccionados, según las cinco áreas de acción propuestas por la OMS para los Estados Miembros y actores sociales (3): identificación de la evidencia, trasmisión de la ciencia y el conocimiento, acciones amplificadas, cuantificación del impacto, y coordinación y gobernanza.

**CUADRO 1.** Acciones emprendidas en los países analizados en respuesta a las cinco áreas de acción propuestas por la Organización Mundial de la Salud^a^

País (fecha del primer caso)	Identificación de la evidencia	Transmisión de la ciencia y el conocimiento	Acciones amplificadas	Cuantificación del impacto	Coordinación y gobernanza	Fuentes
Argentina (1 de marzo del 2020)	- Plataforma *CONFIAR*, de la Secretaría de Medios y Comunicación Pública para combatir la infodemia	- Replicación por medios de comunicación nacionales de "Consejos para la población acerca de los rumores sobre el nuevo coronavirus", de la Organización Mundial de la Salud - Comunicados para desmentir noticias falsas sobre la vacuna contra la COVID-19^b^	- *Cuidar*, aplicación del Ministerio de Salud de la Nación para la prevención y el cuidado de la ciudadanía frente a la pandemia de COVID-19	- Evaluación de informaciones falsas en YouTube	- Se consideró la aplicación del Artículo 211 del Código Penal a los casos de difusión de noticias falsas	11-14
Brasil (25 de febrero del 2020)	- No se identificaron	- No se encontraron	- Canal del Ministerio de Salud por WhatsApp para denunciar "información viral" sobre la COVID-19	- No se ha identificado	- Se aprueba el proyecto de ley 2630/2020 “Ley Brasileña de Libertad, Responsabilidad y Transparencia en Internet”	15, 16
Chile (3 de marzo del 2020)	- Informe de cifras nacionales en páginas oficiales	- Traducción del conocimiento, foros y debates por parte de centros de educación superior	- No se encontraron	- Evaluación de informaciones falsas en YouTube	- Proyecto de ley para sancionar la propagación de noticias falsas en períodos de crisis sanitaria	17-20
Colombia (6 de marzo del 2020)	- Informes diarios del Instituto Nacional de Salud	- Foros y debates por parte de centros de educación superior - Programa televisivo *Prevención y Acción*	- *Colombiacheck* y otros actores reactivan *RedCheq*^c^ - Desarrollo de la aplicación gubernamental *coronaapp*	- Evaluación de informaciones falsas en YouTube	- Página coronaviruscolombia.gov.co enlazada a un centro de la policía y una línea telefónica dedicada - Proyecto de ley para prohibir la creación o utilización de cuentas falsas o anónimas en las redes sociales, que difunden noticias falsas, o generen confusión o pánico	21-23
México (17 de febrero del 2020)	- Página de verificación de la información https://www.infodemia.mx/ - Informe “Noticias falsas y su impacto en el derecho a la libertad de expresión”	- Campaña *Verified* (Verificado)^d^ para combatir las noticias falsas relacionadas con la COVID-19	- Aplicación *COVID-19MX*, de la Secretaria de Salud	- Evaluación de informaciones falsas en YouTube - Evaluación de canales de difusión de noticias falsas en YouTube y redes sociales^e^	- No hay legislación específica y no se han registrado sanciones a particulares o medios de comunicación por parte de la Secretaría de Gobernación	24-27
Perú (6 de marzo del 2020)	- *Handbook COVID-19 Perú*, portal que brinda información relevante y fiable - Organizaciones “chequeadoras”, que combaten la desinformación	- Alfabetización sanitaria en los colegios - Presencia de autoridades de salud pública en las redes sociales	- Hashtag “Don't Spread#noticiasfalsas” por el Ministerio de Salud - Suspensión de cuentas que difunden noticias falsas en Twitter - Eliminación de curas falsas para la COVID-19 en eBay y Amazon	- Evaluación de informaciones falsas en YouTube	- Se establecieron sentencias de prisión para quienes creen o difundan información falsa - Se actualizaron en 2021 los Artículos 315A y 438 del Código Penal^f^	28-32

***Fuente:*** elaborado por los autores.

a Ver referencia 3.

b Elaborada por la Sociedad Argentina de Inmunología, el Instituto de Medicina Experimental y el Centro de Investigación en Bioquímica Clínica e Inmunología.

c RedCheq es una iniciativa de alcance nacional mediante la cual periodistas, medios de comunicación, universidades y organizaciones civiles aliadas combaten la información falsa, engañosa e inexacta sobre la COVID-19.

d Creado y operado por la Organización de las Naciones Unidas, el Centro de Información de las Naciones Unidas para México y el Sistema Público de Radiodifusión del Estado Mexicano.

e YouTube: 10,9%; redes sociales: 82%; de ellas, Whatsapp: 16%; Facebook: 13%; Twitter: 12%.

f Se incluye esta acción, aunque se hizo efectiva fuera del marco temporal que recoge esta investigación.

En las bases de datos de artículos publicados, se realizaron búsquedas específicas por cada país y las palabras clave “COVID-19”, “noticias falsas”, “redes sociales”, además de palabras representativas de las áreas de acción de la OMS: “medios de comunicación”, “transmisión de conocimiento”, “campañas educativas”, “coordinación y gobernanza”, “leyes”, “sentencias”, “decretos”, “acciones gubernamentales” e “informes”, con sus equivalentes en inglés.

La literatura gris se exploró mediante el motor de búsqueda Google con las palabras clave ya descritas separadas por comas y el nombre de cada país; aunque estudios anteriores muestran que las personas no van más allá de la tercera página de búsqueda (37, 38), para esta investigación se tomaron en cuenta las entradas de las primeras cinco páginas.

Para captar más información, se empleó la estrategia de búsqueda conocida como bola de nieve en las referencias y los enlaces sugeridos, tanto en las bases de datos de artículos publicados como en la búsqueda en la literatura gris.

A partir de la información colectada, se evaluó críticamente el cumplimiento de las acciones propuestas por la OMS y su relación con los hallazgos obtenidos en el análisis descriptivo.

Debido a la naturaleza y las fuentes de los datos, no se realizaron análisis de significación estadística y no se buscó determinar la asociación entre la mortalidad y las noticias falsas.

## RESULTADOS

**FIGURA 1.**
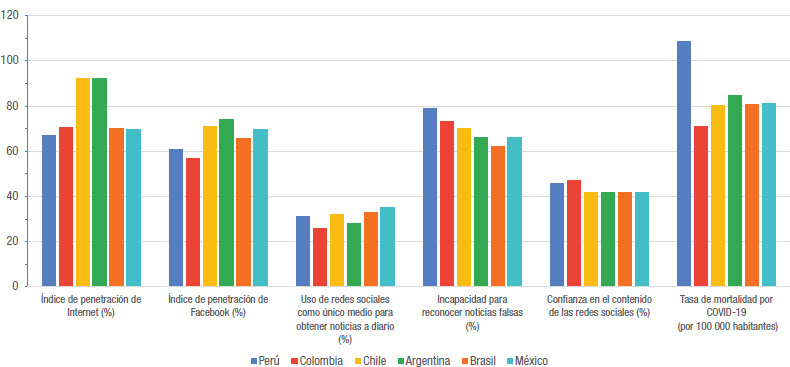
Caracterización de los países analizados, según las variables de estudio, noviembre del 2020

Según la información recolectada, Chile y Argentina fueron los países con los mayores índices de penetración de Internet (92,4% y 92,0%, respectivamente) y Facebook (70,9%, 73,9%, respectivamente), y también hacen un considerable uso de las redes sociales como único medio para la obtención de noticias a diario (32,0% y 28,0%, respectivamente); Brasil y Colombia mostraron un comportamiento intermedio según el índice de penetración de Internet y tuvieron índices de penetración de Facebook relativamente bajos, en comparación con los demás países analizados. México presentó el uso más alto de redes sociales (35%), mientras Perú (79,0%) y Colombia (73,0%) presentaron los mayores valores del índice de incapacidad para reconocer noticias falsas y los menores índices de penetración de Facebook (60,7% y 56,9%, respectivamente) (figura 1).

***Fuente:*** elaborado por los autores.

De los países analizados, Perú —cuya población presentó el mayor porcentaje de incapacidad para reconocer noticias falsas (79,0%) y que fue el segundo con mayor confianza en el contenido de redes sociales (46,0%)— tuvo la mayor mortalidad por COVID-19 (108,7 por 100 000 habitantes) (figura 1).

Al evaluar la incapacidad de la población para reconocer las noticias falsas, se observó un patrón más favorable en Brasil (62,0%), Argentina (66,0%) y México (66,0%) que, además, tuvieron tasas de mortalidad más bajas (entre 81 y 85 por 100 000 habitantes) que en Perú. Contradictoriamente, Colombia (73,0%) y Chile (70,0%) presentaron alta incapacidad para reconocer noticias falsas y baja mortalidad por COVID-19 (71 y 80 muertes por 100 000 habitantes, respectivamente).

Con la excepción de Brasil, donde las acciones del gobierno han sido limitadas, en los restantes cinco países analizados se cuantificó el impacto de la pandemia y se implementaron acciones encaminadas a controlar la infodemia en sus territorios, ya fueran promovidas nacionalmente como internacionalmente (como la campaña *Verified,* en México). En estas acciones participaron actores políticos, la comunidad científica y la población general (cuadro 1). Excepto en Chile, en los países analizados también se realizaron acciones en el campo de las tecnologías de la información y las comunicaciones para dar seguimiento a los casos e informar a la comunidad. En Brasil, Chile, Colombia y Perú se aprobaron o propusieron leyes dirigidas a prevenir la creación y difusión de noticias falsas (cuadro 1).

## DISCUSIÓN

Es un hecho ampliamente reconocido que el aumento en la generación y la propagación de informaciones falsas, engañosas e inexactas, y la dificultad para identificarlas en las redes sociales y los servicios de mensajería instantánea constituyen dos de los mayores obstáculos para el éxito de las acciones de salud pública (39).

En el presente estudio se evidenció una baja capacidad para reconocer las noticias falsas en más de la mitad de la población en los seis países evaluados. Igualmente, se observó que los países con mayor confianza en el contenido de las redes sociales tenían, por lo general, altas tasas de mortalidad, aunque esto no indica que haya una relación causa-efecto entre ambos fenómenos. Se observó que la población de Perú presentó un elevado uso de redes sociales y el mayor porcentaje de incapacidad para reconocer noticias falsas; coincidentemente, ese país presentó la mayor tasa de mortalidad por COVID-19 (34).

Perú fue el primer país en implementar acciones de orden político para controlar las noticias falsas, tanto mediante sentencias —que podían llegar a penas de prisión— como por la utilización por el Ministerio de Salud de las redes sociales para divulgar la información oficial (28). Por su parte, Argentina utilizó las leyes que ya tenía vigentes para castigar el uso inadecuado de la información y evitar la propagación de información falsa en su territorio; además, dio una rápida respuesta mediante las tecnologías de la información y las comunicaciones para orientar a la población y vigilar la calidad de lo que se difunde (28-31).

Los proyectos de ley aprobados o presentados en los congresos legislativos de Brasil, Chile, Colombia y Perú se dirigieron a penalizar las malas conductas relacionadas con la generación y transmisión de informaciones falsas o engañosas sobre la pandemia. También se realizaron acciones de alfabetización sanitaria encausadas por instancias gubernamentales y políticas con el apoyo de la comunidad científica, las redes sociales e instituciones educativas. En este esfuerzo también participaron comunidades “chequeadoras”, es decir, sitios en la Web, blogs, centros, asociaciones (como Colombiacheck, en Colombia) y expertos dedicados a la verificación de los hechos y las noticias circulantes en las redes sociales (como la Sociedad Argentina de Inmunología, el Instituto de Medicina Experimental, el Centro de Investigaciones en Bioquímica Clínica e Inmunología, todos de Argentina), instancias creadas con el objetivo de fortalecer el debate público e identificar informaciones falsas (15, 17-21, 23, 29). México, a pesar de no poseer políticas o leyes que sancionen la distribución de noticias falsas, ha realizado esfuerzos para la verificación de la información (24-27).

En Brasil, es difícil evaluar el papel desempeñado por las noticias falsas y el comportamiento de la mortalidad, debido a que el manejo de la pandemia se ha caracterizado por la escasa implementación de acciones políticas y gubernamentales, problemas de gobernanza, cambios de ministros de salud (40) e insuficiente respuesta a la pandemia en general, lo que ha llevado al país a estar entre los de mayor incidencia y mortalidad en el mundo (41, 42). Algo similar se puede decir de Colombia, donde evaluaciones realizadas sobre los modelos de vigilancia de salud pública han generado múltiples críticas (43).

Se debe resaltar el esfuerzo realizado en Chile y Perú, donde se implementaron acciones gubernamentales dirigidas a evitar la propagación de informaciones falsas, engañosas o inexactas, y mitigar sus efectos durante la crisis sanitaria.

Al analizar estos resultados se deben tener en cuenta algunas debilidades y limitaciones. En primer lugar, el uso de datos agrupados, su falta de sincronismo y el enfoque ecológico del presente estudio impidieron utilizar técnicas de ajuste que hubieran permitido alcanzar resultados más robustos, establecer asociaciones y realizar inferencias individuales en los países analizados. En segundo lugar, al basar el análisis y la evaluación en la información publicada se pudo haber generado un sesgo por no incorporar información relevante que no está disponible al público —como planes de contingencia gubernamentales— o informes académicos con hallazgos negativos sobre las políticas implementadas por los diferentes países. Esto se intentó controlar mediante la búsqueda en la llamada literatura gris en varios portales de cada país.

A pesar de estas limitaciones, este estudio describe el comportamiento de la población de seis países de América Latina frente a la información suministrada en redes sociales y el consumo de noticias falsas, teniendo como contexto de fondo la mortalidad por COVID-19. Las variables analizadas en este estudio pueden formar parte de los múltiples factores que afectan a las tasas de mortalidad por COVID-19 y constituyen un reto importante, dado que surge como un problema de mayor envergadura que lo habitualmente registrado en otras pandemias. Las variables analizadas en este estudio deben entenderse como un reto importante a enfrentar en el campo de la infodemiología debido a las dinámicas propias de los sistemas de comunicación actuales y los nuevos problemas inherentes a estos sistemas, también diferentes a lo visto en pandemias pasadas.

Es imperativo formar a la población en general para que sea capaz de evaluar la calidad y la veracidad de la información que circula en los canales digitales y tome conciencia de las implicaciones que tiene para la salud pública la propagación de contenidos falsos. Se requieren estudios que incorporen variables más específicas y métodos más robustos para determinar el grado de impacto de las noticias falsas en cada país, así como la incorporación de modelos más amplios que contemplen áreas diferentes a las evaluadas por el presente estudio.

## Declaración.

Las opiniones expresadas en este manuscrito son únicamente responsabilidad de los autores y no reflejan necesariamente los criterios y las políticas de la *Revista Panamericana de Salud Pública / Pan American Journal of Public Health* y/o de la Organización Panamericana de la Salud.
